# Modeling the influence of basketball players’ offense roles on team performance

**DOI:** 10.3389/fpsyg.2023.1256796

**Published:** 2023-09-08

**Authors:** Ruobing Chen, Mingxin Zhang, Xiao Xu

**Affiliations:** ^1^China Basketball College, Beijing Sport University, Beijing, China; ^2^Athletic Performance College, Shanghai University of Sport, Shanghai, China; ^3^College of Physical Education, Dalian University, Dalian, China

**Keywords:** team performance, cluster analysis, Chinese Basketball Association, play type, player roles

## Abstract

This study aimed to (1) use the clustering method to build a classification model based on the play-type data of basketball players, to classify native and foreign players into different offensive roles; (2) use the clustered offensive role model to investigate how different offensive roles influence team performance. The sample was drawn from 20 teams spanning five seasons (2017–2021) in the Chinese Basketball Association, comprising 823 native and 228 foreign players. The clustering results obtained fourteen offensive roles for native players and five for foreign players. Subsequent analyses revealed that the offensive roles of two native player clusters, namely N6 Spot-up Wings who Attack (OR = 3.281, *p* < 0.05) and N13 Bigs who Cut to the Rim (OR = 4.272, *p* < 0.05), significantly influenced team performance. Conversely, no significant impact was observed for foreign players. The findings of this study offer novel insights into player dynamics and offer coaches a fresh perspective on team composition.

## 1. Introduction

The primary objective of sports performance analysis is to enhance the comprehension of sports and consequently guide decision-making processes for individuals aiming to improve their athletic performance (O’donoghue, 2009). Moreover, the dynamic and intricate nature of sports requires a careful analysis through quantitative measurement techniques (Hughes and Franks, 2007). In earlier years, the book ‘Moneyball’ discussed the use of advanced statistical analysis to evaluate baseball players’ performance and worth. This challenged established scouting strategies that typically depend on subjective judgment and outdated data metrics (Lewis, 2004). Previous research has primarily concentrated on retrospective static analyses utilizing historical data and statistical methods to evaluate the performance of individual players or teams (Gomez-Ruano et al., 2020). For instance, distinguishing between winning and losing teams (Gómez et al., 2006; Çene, 2018), elucidating the significance of contributions made by starters and substitutes during games (Gòmez et al., 2009), analyzing variances in physiological indicators among players in distinct positions (Teramoto and Cross, 2018; Zarić et al., 2020), investigating gender disparities (Gómez et al., 2013), and conducting cross-sectional comparisons of performance data across different leagues (Sampaio et al., 2006).

However, over the past decade, researchers have increasingly adopted dynamic and intricate analysis techniques, especially in developing performance models (Gomez-Ruano et al., 2020). These advanced techniques have proven valuable in processing and analyzing the wealth of available sports data, facilitating the construction of more accurate and reliable performance models (Haghighat et al., 2013). For instance, decision trees have been utilized by researchers to identify patterns in player behavior and performance (Willoughby and Kostuk, 2005; Morgan et al., 2013). Logistic regression has been employed to model the probability of specific game outcomes or player actions (Zdravevski and Kulakov, 2009; Igiri and Nwachukwu, 2014). Support vector machines (SVM) have been used for tasks such as player classification or predicting player attributes (Mustafa et al., 2017; Pai et al., 2017). Random forests have shown promise in capturing complex relationships within sports data and making accurate predictions (Lin et al., 2014; Knoll and Stübinger, 2020). In addition to machine learning models, deep learning approaches, like neural networks, have gained popularity in sports analytics due to their ability to automatically learn and extract intricate patterns from large datasets (Haghighat et al., 2013). These models have demonstrated success in tasks such as player trajectory prediction and play sequence analysis (Kahn, 2003; Ivanković et al., 2010). Moreover, Ranking models, such as Elo Rating, TrueSkill scores, and PageRank algorithms, have been commonly used to assess player or team performance relative to their peers (Barrow et al., 2013; Ibstedt et al., 2019; Kovalchik, 2020). Furthermore, sports research has extensively explored the application of both quantitative and qualitative models. Rule-based models offer interpretability and have been employed to derive decision-making strategies in various sports contexts (Zdravevski and Kulakov, 2009; Trawiński, 2010). Time series models are often used to capture temporal dependencies in sports data, enabling trend analysis and forecasting player performance over time (Yong et al., 2020). Clustering models have been utilized to group players or teams based on similar characteristics, facilitating player profiling and performance comparison (Patel, 2017; Mateus et al., 2020). This combination of analytic techniques, encompassing both quantitative and qualitative variables, provides the foundation of data and information. Importantly, the synergy between these two types of data enables researchers to understand and predict athlete performance more comprehensively, offering valuable insight into the multifaceted drivers of sports performance (O’Donoghue, 2014).

Assessing the critical performance indicators of players and teams is an equally important aspect of sports analysis (Sarlis and Tjortjis, 2020). Previously, basketball data expert Dean Oliver presented a pioneering understanding of data analysis (Oliver, 2004), prompting scholars to subsequently develop diverse metrics for evaluating the game from multiple viewpoints (Cooper et al., 2009; Metulini and Gnecco, 2022). Given that the intricacy of match performance stems from the unique contextual setting in which each match takes place, situational elements like location, opponent, and stage of play exert behavioral influences on the performance of athletes and sports teams (McGarry et al., 2013). Consequently, researchers must pay meticulous attention to the impact of contextual variables when constructing statistical models and investigating critical competition indicators within diverse competitive contexts and conditions, thereby establishing a standardized profile of technical performance during competitions (Hughes et al., 2001; Zhang et al., 2017). Certain studies have consolidated performance indicators across various sports, encompassing game, tactical, technical, and biomechanical indicators on a macro level (Hughes and Bartlett, 2002). Basketball scholars have refined these indicators by introducing Team Performance Indicators (TPI, Yu et al., 2008), which have been instrumental in investigating different facets of basketball.

Basketball is a team sport where success primarily hinges on integrating players with complementary skills (Katzenbach and Smith, 2015). The study of team composition has consistently regarded player positions as a crucial factor, traditionally determined by height and weight considerations (Deman et al., 2001). In particular, taller or more robust players are often stationed near the basket, while smaller players typically exhibit more significant activity on the perimeter (Latin et al., 1994; Ostojic et al., 2006). The conventional approach categorizes players into five positions: Point Guard, Shooting Guard, Small Forward, Power Forward, and Center (Trninić et al., 1999). However, as players develop physically and technically, they can assume multiple roles on the court. Consequently, the traditional method tends to oversimplify the skill sets of high-level players, resulting in suboptimal utilization of team resources (Cheng, 2017). Scholars commonly employ cluster analysis in unsupervised learning to study player positions. Examples encompass hierarchical clustering (Whitehead, 2017a), topology (Alagappan, 2012), K-means and model-based clustering (Kalman and Bosch, 2020), and Gaussian mixture clustering (Wang et al., 2022). Additionally, discriminant analysis (DAs) has demonstrated its suitability for predicting player positions (Pion et al., 2018). However, the studies mentioned above primarily rely on basic statistics, such as box scores, which have inherent limitations (Shea and Baker, 2013). For instance, high scoring can be attributed to a player’s shooting technique or the ability of teammates to create favorable shooting opportunities. Similarly, the frequency of defensive rebounds can be influenced by rebounding technique or the number of missed shots by opponents (Remmert, 2003; Christmann et al., 2018). Certain researchers have investigated player behavior by considering interactive information, such as passing and pick-and-roll actions, consistently demonstrating that cooperative play between players yields superior results compared to individual actions (Courel-Ibáñez et al., 2013; Gómez et al., 2015; Marmarinos et al., 2016). Consequently, the study of interactive information data can illuminate the technical and tactical aspects and is more favorable than traditional statistics for analyzing and evaluating athletic performance, as well as assisting coaches in decision-making (Christmann et al., 2018).

Synergy, a sports data science company, offers play-type data and game footage access. They also provide advanced visualization tools for coaches and analysts to analyze opponents’ playing styles, monitor player development over time, and gain insights into player and team performance (Božović, 2021; Fu and Stasko, 2022). There are 11 standardized play types in offense, such as spot-up, transition, isolation, off-screen, and so on (Božović, 2021). Basketball offensive tactics are aimed at creating optimal shot selection within a 24-s period. The interplay of gaining separation from the defender(s) (e.g., to shoot or receive a pass) and gaining proximity (for a screen, handoff, cut, or drive) is characteristic of the basketball game (Christmann et al., 2018). These patterns or actions are designed by the coaches, heavily practiced by the players, and then repeated in the game. Understanding how players and teams create successful scoring opportunities is, therefore, critical both practically and theoretically (Matulaitis and Bietkis, 2021). For example, poor pick-and-roll use is recognized as a significant cause of team failure (Vaquera et al., 2013); the most effective finishing moves in the Euroleague are isolation, pick-and-roll, spot-up, and cut (Matulaitis and Bietkis, 2021), while the finishing actions that contribute the least to scoring are off-screen and hand-off (Zukolo et al., 2019). The applicability of these data presents an excellent tool for detailed analysis and creating scout reports (Božović, 2021). Scholars have utilized this data to investigate team styles, player roles, and the influence of various game types on team performance (McBasketball, 2017; Božović, 2021), thereby providing basketball statisticians with a novel perspective for player observation. It is worth noting that player play type is a relatively understudied topic, with minimal research focused on it. Moreover, such studies are particularly scarce for high-level Asian leagues. Therefore, building on prior research, we postulate that classifying players’ play-type data will reveal contrasting offensive roles between native and foreign players, which, in turn, will have distinct impacts on team performance.

Given the above considerations, the aims of this study are as follows: (1) use the clustering method to build a classification model based on the play-type data of basketball players, to classify native and foreign players into different offensive roles; (2) use the clustered offensive role model to investigate how different offensive roles influence team performance.

## 2. Materials and methods

### 2.1. Sample and variables

The research data was obtained from the sports data website Synergy,^1^ and we gained the license to use play-type related data and video. Table 1 presents the category of 11 play types in basketball, these indicators are considered standardized and cover all scoring attempts in the game of basketball, and they were able to translate the team’s tactical decisions into countable data (McBasketball, 2017; Božović, 2021). Play-type data was collected for players from 20 teams during five CBA seasons, from 2017 to 2022, encompassing a total of 4,475 games including regular season, playoff qualifying rounds, and playoffs. The raw data consisted of 1,454 native players and 265 foreign players. To ensure the dependability of the clustering and subsequent analysis, players who had less than 3 % of the team’s total scoring attempts were excluded. Consequently, our final sample included 823 native players (*n* = 823) and 228 foreign players (*n* = 228).

**Table 1:** K-means clustering-related play-type indicator.

Play-type	Description
Spot-up	When the possession-ending event is a catch-and-shoot or catch-and-drive play.
Pick-and-roll ball-handler	A screen is set on the ball handler’s defender out on the perimeter. The offensive player can use the screen or go away from it, and as long as the play yields a possession-ending event, it is tagged as a pick and roll.
Transition	When the possession-ending event comes before the defense sets following a possession change and a transition from one end of the court to the other.
Cuts	An interior play where the finisher catches a pass while moving toward, parallel to, or slightly away from the basket. This will include the back screen, flash cuts, and times when the player is left open near the basket.
Pick-and-roll man	When a screen is set for the ball handler, the setter receives the ball for a possession-ending event. This action can include: pick and rolls, pick and pops, and the screener slipping the pick.
Post-up	When an offensive player receives the ball with their back to the basket and is less than 15 feet from the rim when the possession-ending event occurs.
Off-screen	Identifies players coming off of screens (typically downs screens) going away from the basket toward the perimeter. This includes curl, fades, and flare.
Putbacks	When the rebounder attempts to score before passing the ball or establishing themselves in another play type.
Isolation	When the possession-ending event is created during a “one-on-one” match-up, the defender needs to be set and have all of his defensive options at the initiation of the play.
Hand-off	The screen setter starts with the ball and hands the ball to a player cutting close by. This enables the player handing the ball off to effectively screen off a defender creating space for the player receiving the ball.
Miscellaneous	When the action does not fit any of the other play types. This includes but is not limited to last-second full-court shots, fouls in the backcourt, and errant passes not out of a different play type.

### 2.2. Statistical analysis

The initial step involved K-means clustering in categorizing native and foreign players into distinct groups based on their play type data (Zhang et al., 2016; Patel, 2017). The elbow method was employed to determine the optimal number of clusters for the two groups. The clustering results were assessed using the silhouette coefficient (Zhou and Gao, 2014), which ranges between-1 and 1. A higher value closer to 1 indicates superior clustering results, while a negative value indicates poor results. After the clustering results were obtained, the dataset was depersonalized using the t-SNE method, and the results were then visualized (Van der Maaten and Hinton, 2008; Patel, 2017). The process above was implemented using the scikit-learn machine learning framework in Python.

In the second step, non-parametric tests were employed to examine the potential differences in the number of distinct offensive roles between teams that reached the semi-finals and those that did not. Effect sizes (ES) were calculated using Cohen’s d, and their interpretation followed the established criteria: 0.10 denoted a small effect, 0.30 indicated a medium effect, and 0.50 marked a large effect (Volker, 2006). Additionally, we constructed a logistic regression model to investigate the impact of each offensive role on the team’s performance. The number of distinct offensive roles within the team served as the independent variable, while whether the team reached the semi-finals was the dependent variable. Based on their rankings in the season, all teams were categorized as either non-semi-finalists or semi-finalists (code: 0, 1). The threshold for determining statistical significance was set at *α* = 0.05. The above analyses were conducted using IBM SPSS Statistics for Mac, version 24.0 (Armonk, NY: IBM Corp.).

### 2.3. Reliability and validity of data

To establish the validity of the play type data obtained from Synergy, a random selection of five games per season was observed by three analysts with over 5 years of experience in basketball video analysis. This observed data was then combined with the information provided by Synergy to conduct an intra-group correlation analysis using the Two-Way Mixed-Effects Model (Koo and Li, 2016), ultimately showing a high degree of consistency (ICC = 0.99). The local research institutional review board formally approved all procedures.

## 3. Results

### 3.1. Players offensive role definition

**Figure 1:**
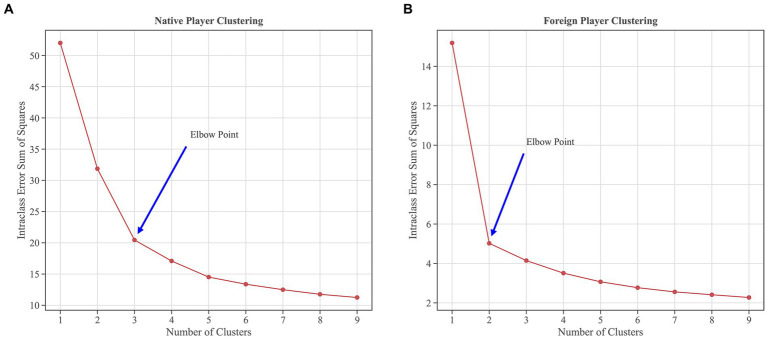
The elbow method determines the optimal number of clusters for a player’s offensive role. Subfigure **(A)** demonstrates a declining inflection point in SSE for native players when the number of clusters is three; for foreign players, Subfigure **(B)** demonstrates a declining inflection point in SSE when the number of clusters is two.

Before clustering, the “elbow method” is employed to determine the optimal number of clusters. The primary concept behind this method involves identifying the inflection point where the sum of squared errors (SSE) within a cluster decline significantly. This inflection point represents the optimal number of clusters (Zhang et al., 2016; Cui, 2020). According to Figure 1, the optimal number of clusters for native players is three, while for foreign players, it is two.

**Figure 2:**
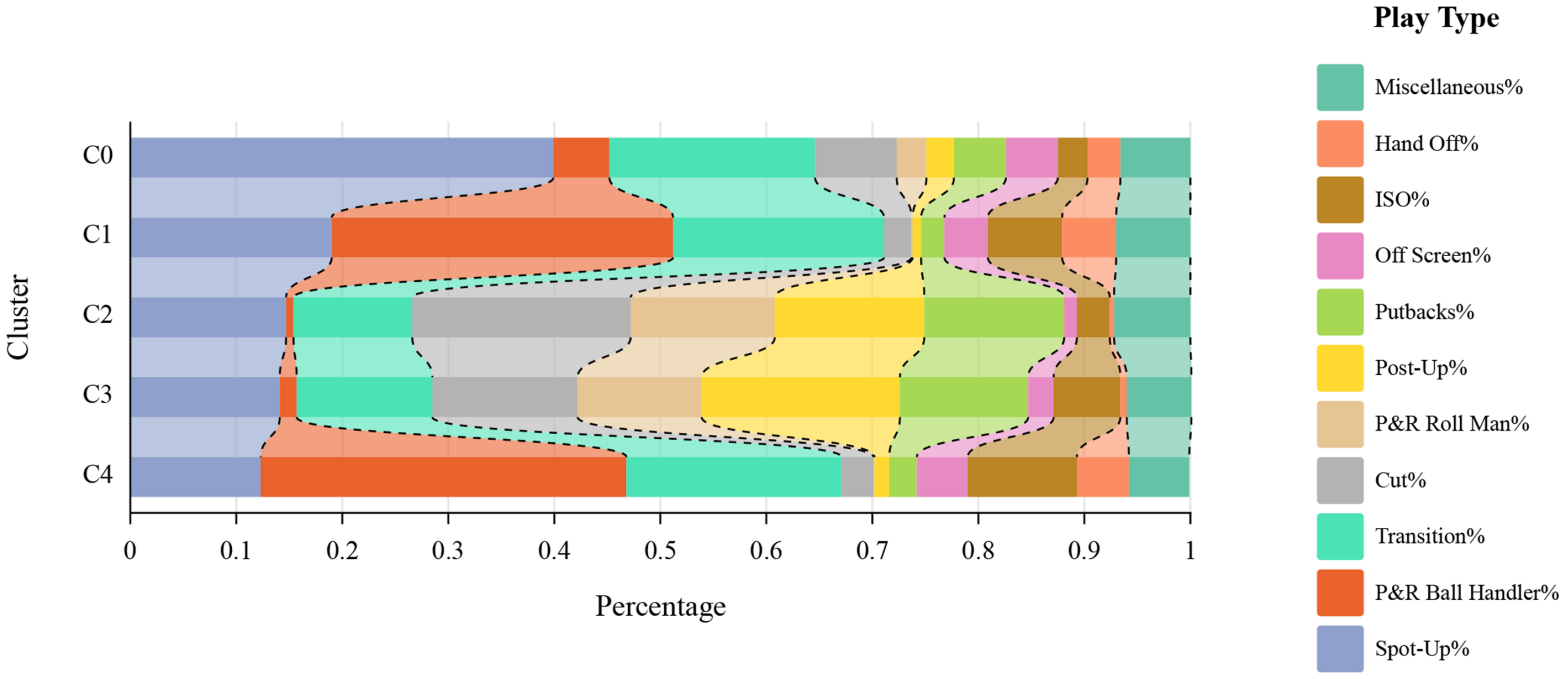
Play-type percentage of different clusters (Macro offensive role).

Based on the play type percentages depicted in Figure 2, the players can be macroscopically categorized as ball-handlers (C1, C4), wings (C0), and big men (C2, C3) (Whitehead, 2017a; Diambra, 2018). The specific definitions of these three types of roles are as follows:

Ball-Handlers: These roles have a high percentage of pick-and-roll ball-handlers and transition.

Wings: These roles have a high percentage of spot-up and transition.

Big men: These roles have a high percentage of cut, pick-and-roll man, post-up, and putbacks.

**Figure 3:**
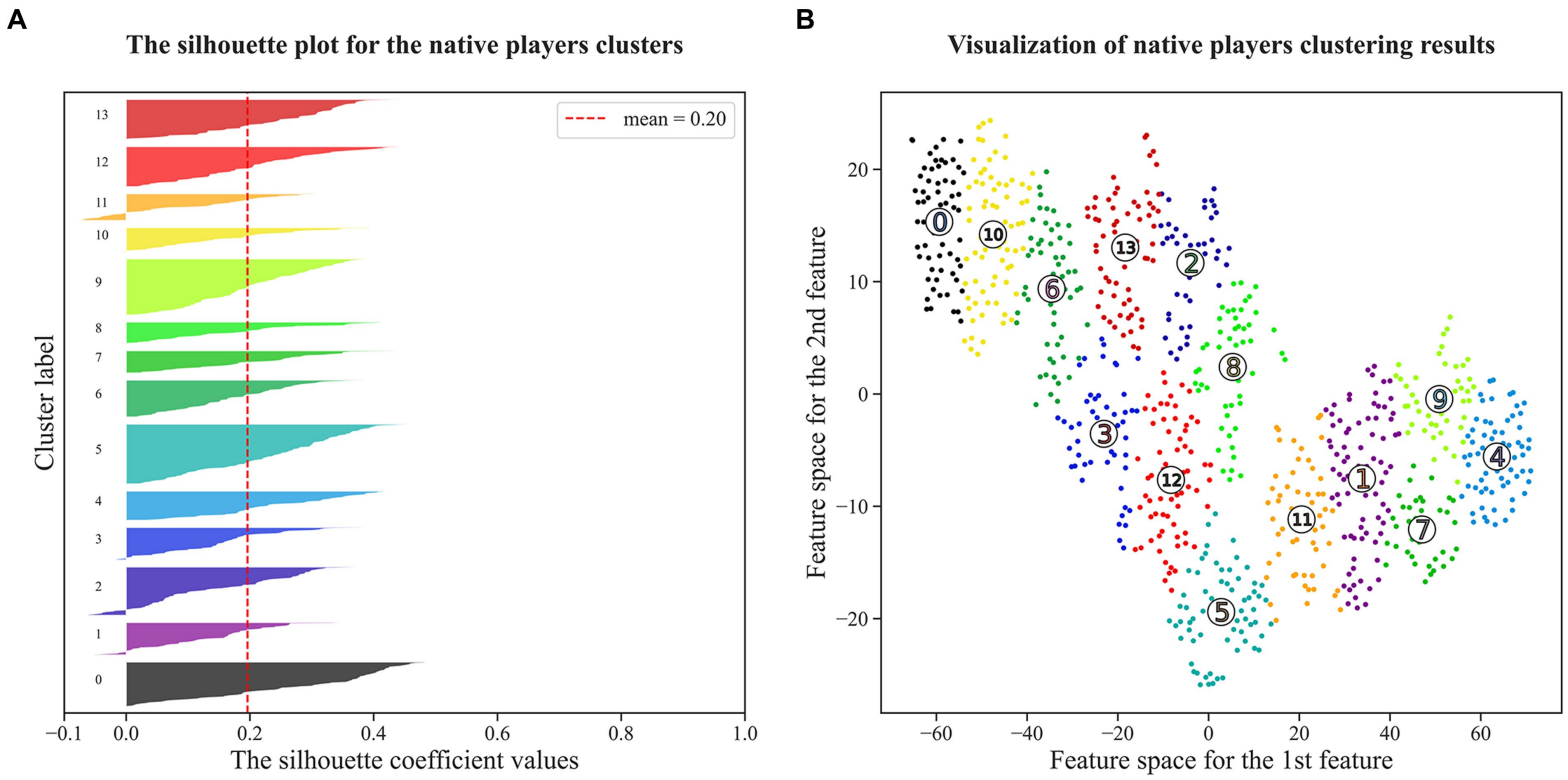
Visualization of clustering results for native players. Subplot **(A)** shows that the silhouette coefficients of all clusters exceed the mean value. Subfigure **(B)** transforms eleven play-type variables into two dimensions by the t-SNE dimensionality reduction technique, and the numbers in Subfigure **(B)** correspond to the Cluster Label in Subplot **(A)**.

**Figure 4:**
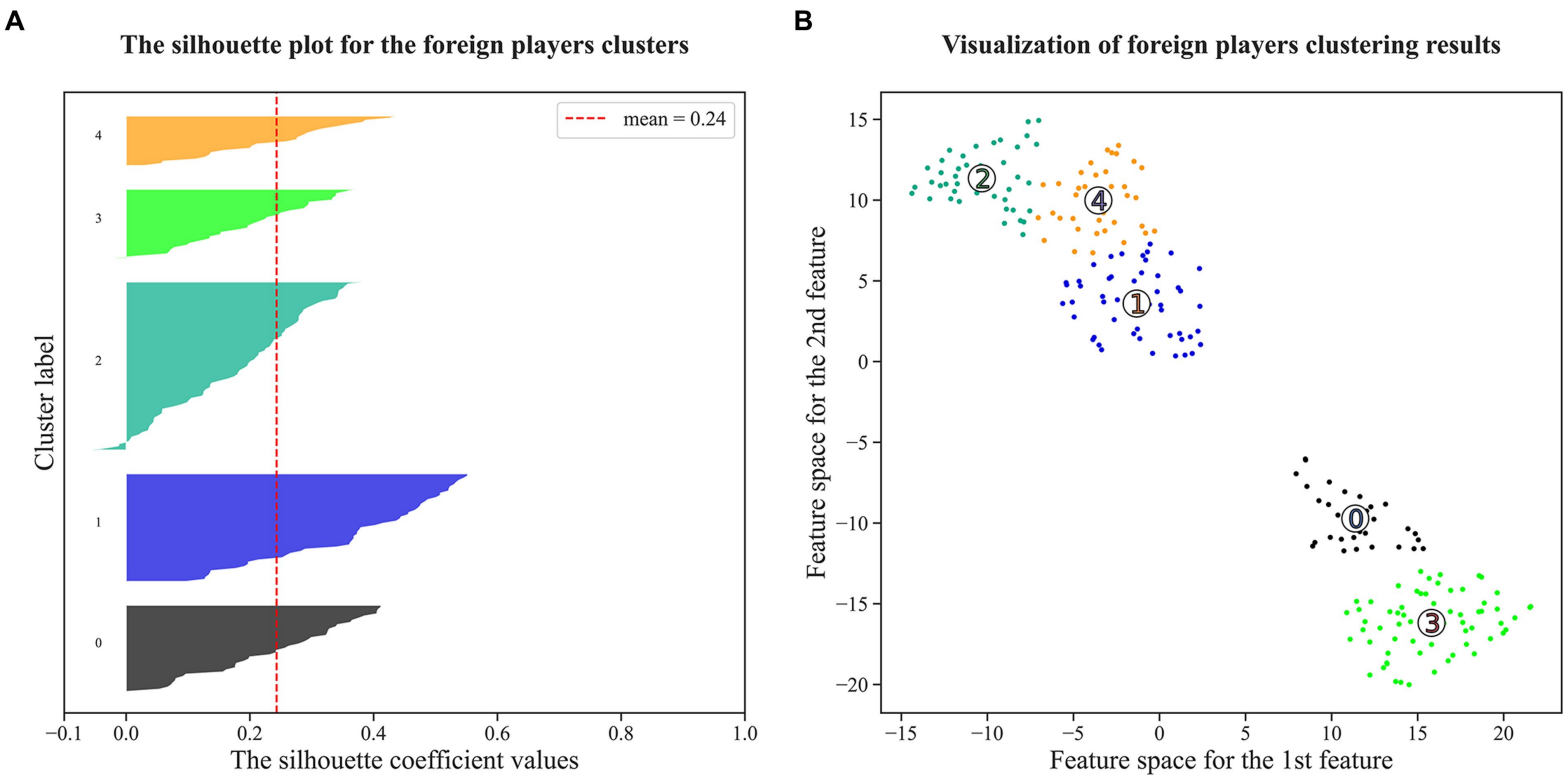
Visualization of clustering results for foreign players. Subplot **(A)** shows that the silhouette coefficients of all clusters exceed the mean value. Subfigure **(B)** transforms eleven play-type variables into two dimensions by the t-SNE dimensionality reduction technique, and the numbers in Subfigure **(B)** correspond to the Cluster Label in Subplot **(A)**.

Nevertheless, in the context of basketball development, limiting the definition to only three roles deviates from the sport’s current developmental trend (Bianchi et al., 2017; Whitehead, 2017a). Consequently, we used K-means clustering to generate fourteen offensive roles (N0-N13) for native players and five (F0-F4) for foreign players. According to Figures 3, 4, the silhouette coefficient calculated for clustering native players is 0.20, indicating a moderate level of both cohesion and separation among the clusters. Put simply, the clusters formed for native players exhibit a certain level of internal similarity while maintaining reasonable distinction from each other. On the other hand, the silhouette coefficient for clustering foreign players is 0.24, indicating an enhanced clustering quality for this group. The clusters formed for foreign players demonstrate improved internal homogeneity and clearer boundaries between them (Zhou and Gao, 2014).

**Figure 5:**
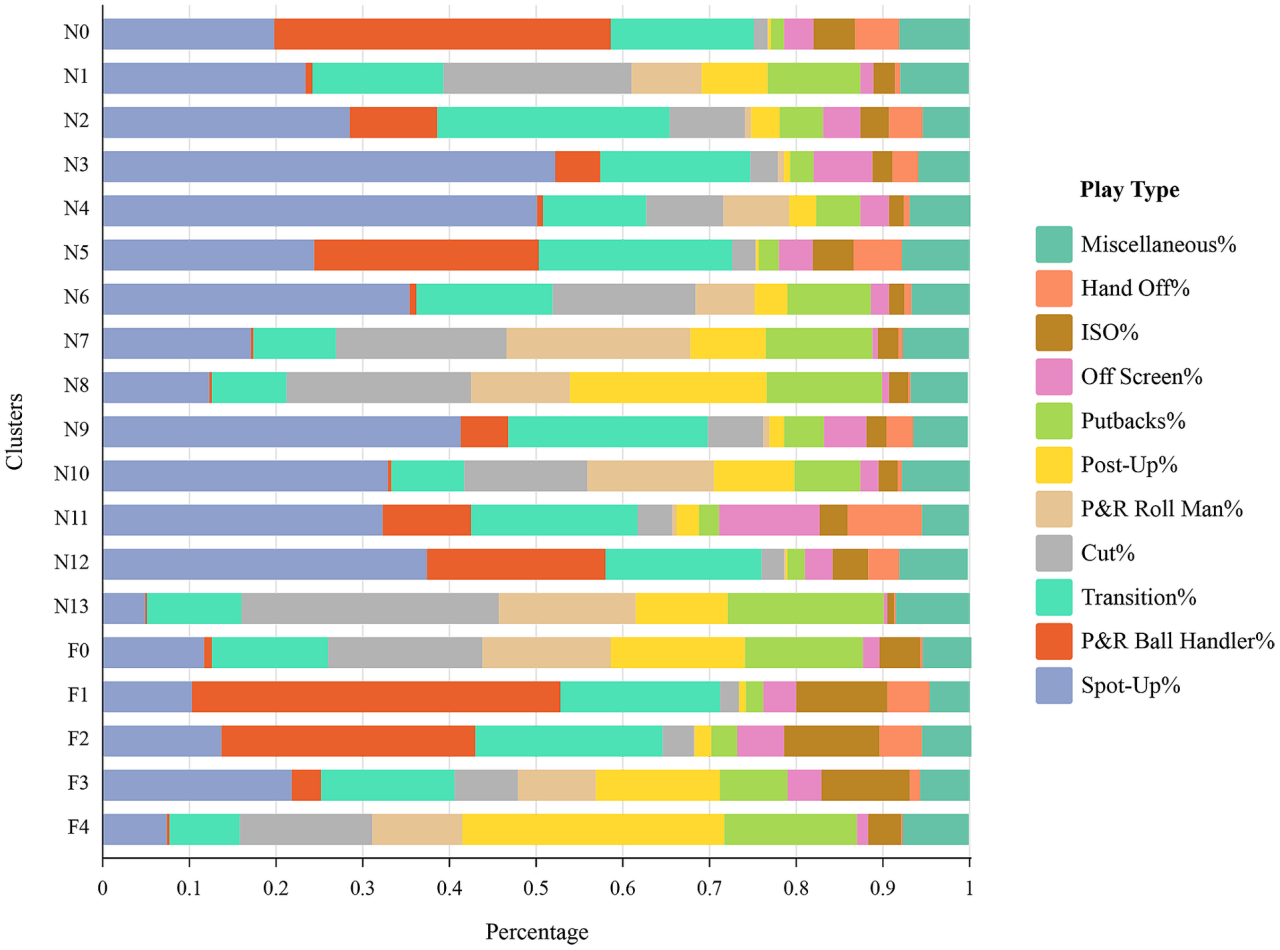
Play-type percentage of different clusters (Specific Offensive roles).

Figure 5 displays the distribution of player clusters based on play type percentages. Similar to other studies that have analyzed player positions and roles using clustering methods, we identify players by observing how they are represented in different types of play data (Lutz, 2012; Wang et al., 2022). The subsequent descriptions outline the offensive roles associated with each offensive role:

N0, “Primary Ball Handlers”: This group consists of native players with the highest percentage of pick-and-roll ball handlers.

N1, “Mobile Bigs who Cut”: This category comprises big men with exceptional mobility and a cutting style of scoring.

N2, “Impact Wings”: These players exhibit the highest percentage of participation in transition offenses.

N3, “Stationary Spot-up Wings”: This offensive role demonstrates the highest percentage of spot-up plays among all players.

N4, “Stationary Tall Wings”: These players exhibit a slightly lower percentage of spot-ups than N3 players but a higher percentage of roll-man plays and putbacks.

N5, “Ball Handlers who Share the Load”: This offensive role demonstrates a slightly lower percentage of pick-and-roll ball-handler than the N0 players.

N6, “Spot-up Wings who Attack”: These players possess a high spot-up percentage but primarily rely on cuts, roll-man, and putbacks to score.

N7, “Bigs who Roll to the Rim”: These players play a significant role in the pick-and-roll offense, frequently rolling to the basket to score after setting a screen.

N8, “Mobile Bigs who Post-up at the Rim”: These players exhibit the highest post-up percentage among native players and actively contribute to pick-and-roll plays and cuts for scoring opportunities.

N9, “Spot-up Wings who Transition”: This offensive role predominantly engages in spot-up and transition plays, while other play types are less prevalent.

N10, “Tall Wings who Pick-and-pop”: These players are characterized by a high spot-up and roll-man percentage, indicating their inclination toward catch-and-shoot plays following a pick.

N11, “Wings who Work Around Screen”: This offensive role excels at scoring by utilizing screens set by teammates on the court, leading to a high off-screen and handoff percentage.

N12, “Ball-handler with Off-ball Duties”: This offensive role exhibits a lower pick-and-roll ball-handler rate than N0 and N5 players but demonstrates a higher spot-up percentage.

N13, “Bigs who Cut to the Rim”: These players exhibit the highest percentage of cuts and putbacks while displaying the lowest percentage of spot-up plays.

F0, “Bigs with Skills Everywhere”: These skilled bigs employ a diverse array of plays to attempt scoring, including posting up inside, spotting up outside, and executing cuts or rolls to the basket.

F1, “Domineering Ball Handlers”: These players possess the highest percentage of pick-and-roll ball handlers and exhibit much isolation plays.

F2, “Wings who Initiate Offense”: These players demonstrate a particular percentage of pick-and-roll ball-handling and isolation plays. Despite not being the primary ball handlers on the court, they can initiate attacks with the ball.

F3, “Ball Stopping Mobile Bigs”: This offensive role is distinguished by a high percentage of spot-ups and isolation plays, often resulting in individual finishes for possessions.

F4, “Bigs with Skills in the Post”: This offensive role demonstrates the highest post-up percentage, indicating a greater emphasis on low-post offense.

### 3.2. Analysis of the influence of offensive roles on team performance

We initially examined if there existed differences in the diversity of offensive roles within the teams. Non-parametric test results (Table 2) indicate that categories N10 Tall Wings who Pick-and-pop (*p* < 0.01, ES = 0.84), N13 Bigs who Cut to the Rim (*p* < 0.05, ES = 0.62), and F4 Bigs with Skills in the Post (*p* < 0.05, ES = 0.67) exhibited significant disparities among the teams that advanced to the semi-finals and those that did not.

**Table 2:** Non-parametric test of the number of different offensive roles.

Clusters	Whether to reach the semi-finals(M ± SD)		Mann–Whitney U	Mann–Whitney z	*p*	ES
	No (*n* = 79)	Yes (*n* = 20)				
N0	0.68 ± 0.96	0.85 ± 1.04	724	−0.635	0.525	0.17
N1	0.51 ± 0.64	0.60 ± 0.68	731	−0.582	0.56	0.14
N2	0.75 ± 0.91	0.90 ± 1.17	760	−0.284	0.776	0.15
N3	0.51 ± 0.71	0.60 ± 0.75	735.5	−0.544	0.586	0.13
N4	0.48 ± 0.68	0.45 ± 0.51	770.5	−0.197	0.844	0.05
N5	1.00 ± 0.97	0.80 ± 0.62	733	−0.528	0.597	0.25
N6	0.58 ± 0.79	0.65 ± 0.49	675	−1.122	0.262	0.10
N7	0.35 ± 0.64	0.40 ± 0.50	712	−0.853	0.394	0.08
N8	0.37 ± 0.70	0.30 ± 0.47	788	−0.022	0.982	0.11
N9	0.87 ± 0.76	1.00 ± 0.80	727.5	−0.59	0.555	0.16
N10	0.49 ± 0.83	0.00 ± 0.00	520	−3.017	0.003^**^	0.84
N11	0.49 ± 0.68	0.20 ± 0.41	616	−1.794	0.073	0.53
N12	0.63 ± 0.85	0.70 ± 0.57	682.5	−1.038	0.299	0.09
N13	0.54 ± 0.68	1.05 ± 0.95	552	−2.286	0.022^*^	0.62
F0	0.42 ± 0.52	0.40 ± 0.60	759	−0.317	0.751	0.03
F1	0.51 ± 0.60	0.55 ± 0.69	776.5	−0.134	0.894	0.07
F2	0.79 ± 0.81	0.85 ± 0.75	735	−0.518	0.604	0.08
F3	0.34 ± 0.53	0.30 ± 0.57	745	−0.49	0.624	0.08
F4	0.29 ± 0.46	0.05 ± 0.22	599.5	−2.237	0.025^*^	0.67

*Means *p* < 0.05, **means *p* < 0.001, ES, Effect Size.

Further, we constructed a logistic regression model using the number of offensive roles and the team’s performance. Table 3 displays the logistic regression model’s indicators. For native players, significantly positive effects were observed for N6 Spot-up Wings who Attack (OR = 3.281, *p* < 0.05) and N13 Bigs who Cut to the Rim (OR = 4.272, *p* < 0.05). The offensive roles of foreign players did not significantly impact the team’s quality.

**Table 3:** Logistic regression analysis of players’ offensive roles.

Clusters	*β*	SE	*z* score	Wald χ^2^	*p*	Odds ratio	CI 95%
N0	−0.123	0.403	−0.305	0.093	0.761	0.884	0.401 ~ 1.950
N1	0.92	0.673	1.367	1.869	0.172	2.509	0.671 ~ 9.381
N2	0.248	0.423	0.586	0.343	0.558	1.281	0.559 ~ 2.934
N3	−0.255	0.659	−0.388	0.15	0.698	0.775	0.213 ~ 2.817
N4	0.399	0.721	0.553	0.306	0.58	1.49	0.363 ~ 6.125
N5	−0.576	0.511	−1.127	1.27	0.26	0.562	0.207 ~ 1.531
N6	1.188	0.586	2.029	4.116	0.042^*^	3.281	1.041 ~ 10.342
N7	0.985	0.816	1.207	1.457	0.227	2.678	0.541 ~ 13.259
N8	1.026	0.969	1.058	1.12	0.29	2.789	0.417 ~ 18.635
N9	0.39	0.555	0.703	0.494	0.482	1.477	0.497 ~ 4.389
N10	−1.675	0.579	−1.957	3.571	0.997	0.383	0.027 ~ l.272
N11	−1.416	0.945	−1.498	2.244	0.134	0.243	0.038 ~ 1.547
N12	−0.009	0.558	−0.016	0.075	0.987	0.991	0.332 ~ 2.960
N13	1.452	0.629	2.31	5.335	0.021^*^	4.272	1.246 ~ 14.649
F0	−0.002	0.75	−0.002	0.068	0.998	0.998	0.229 ~ 4.344
F1	0.669	0.782	0.855	0.731	0.393	1.952	0.421 ~ 9.044
F2	0.3	0.578	0.518	0.269	0.604	1.35	0.434 ~ 4.194
F3	0.315	0.803	0.393	0.154	0.695	1.371	0.284 ~ 6.610
F4	−2.008	1.285	−1.562	2.439	0.118	0.134	0.011 ~ 1.668

Nagelkerke *R*^2^ = 0.468, *β*, Regression Coefficient; SE, Standard Error. *means *p* < 0.05.

## 4. Discussion

The aim of this study was to (1) use the clustering method to build a classification model based on the play-type data of basketball players, to classify native and foreign players into different offensive roles; (2) employ the classified offensive role model to examine the impact of diverse offensive roles on team performance.

Performance modeling is a valuable tool with diverse applications, including predicting game results (Zdravevski and Kulakov, 2009), identifying tactical strategies (Tian et al., 2019), aiding in player draft and recruitment processes (Berri et al., 2011), and evaluating player and team performance (Terner and Franks, 2021). Several studies have utilized this approach to categorize players’ positions and roles and forecast their performance (Rangel et al., 2019; Wang et al., 2022). However, comparative studies between native and foreign players have rarely employed similar approaches. Most studies have consistently demonstrated the superior capabilities of foreign players compared to native players, suggesting their need for a more significant on-court role (Guimarãe et al., 2018; Gasperi et al., 2020). Performance modeling helps mitigate these biases and preconceptions, enabling a fair and data-driven assessment of players from different backgrounds. This is especially crucial for foreign players, as their style and skill sets may differ from native players due to cultural, training, and developmental factors (Ozmen, 2012). Nevertheless, it is essential to recognize the limitations of performance modeling. The reliability of predictions can be influenced by the quality, completeness, and accuracy of the data on which the models rely (Fu and Stasko, 2022). Additionally, basketball is a complex and dynamic game influenced by various situational factors, teamwork, and coaching strategies, which the models may not fully capture. Caution should be exercised by coaches and researchers when interpreting and relying solely on predictions generated by performance modeling, acknowledging the critical role of human judgment and expertise in the decision-making process (Remmert, 2003; Lamas et al., 2015).

To accurately reflect the contemporary dynamics of the sport, it is necessary to reassess the categorization of player roles in modern basketball. Conventional classifications, including point guard, shooting guard, small forward, power forward, and center, no longer encompass versatility and blurred positional boundaries (Latin et al., 1994; Ostojic et al., 2006). Contemporary players possess a broader spectrum of skills and physical conditioning, allowing them to assume multiple roles on the court. This transformation is propelled by the emphasis on versatility, floor spacing, and the ability to create mismatches (Bianchi et al., 2017; Whitehead, 2017b). In their study of the Brazilian professional basketball league, Rangel et al. noted that versatility players showed an upward trend over nine seasons and presented a higher frequency at the power forward and point guard positions (Rangel et al., 2019). Trninić et al. proposed multidimensional criteria for the various positions and roles of the game of basketball, and their findings showed that the ability to play multiple positions is crucial for players at positions 2 and 3, while players at position 1 are more expert players (Trninić et al., 2000). In basketball, certain guards assume the role of ball handlers within their team, which forwards or centers may also take on. Conversely, some guards do not fulfill the ball handler role, and this principle can be extended to other positions on the court (Sailofsky, 2018). This coincides with our findings: our clustering results yielded 8 categories of wings and 7 categories of big men, reflecting the importance of forward players playing multiple roles in the game; there were only 4 categories of ball-handlers, suggesting that combing the offense and creating scoring chances are their main responsibilities. In addition, the study by Rangel et al. noted that the roles of foreign players showed higher diversity (Rangel et al., 2019). In our study, except for F1 players who are required to be the team’s primary ball-handler, the other types of players showed diversity in their scoring styles. Although the diversity of player roles is a growing trend in basketball, having more than three such “glue guys” proved to be detrimental to team performance in the study by Lutz (2012). Therefore, coaches should try to balance the playing positions and tactical roles of players when constructing lineups.

Our study employed the elbow method to categorize players into three macro-level roles: ball handlers, wings, and big men. Previous studies have reported similar findings, corroborating and reinforcing our findings (Bianchi et al., 2017; Whitehead, 2017a). These findings offer valuable insights for analyzing player roles and conducting further research on the evolution of basketball tactics. Subsequent categorization revealed 14 offensive roles for native players and five for international players. Several previous studies have also created specific labels for basketball players’ roles in the game. For example, Alagappan et al. expanded the traditional five positions to 13 (Alagappan, 2012), and Lutz et al. defined players into 10 different clusters (Lutz, 2012). This categorization, however, fails to fully transcend the conventional positional constraints due to the substantial variation in statistical metrics across different player positions. For instance, guards tend to have higher counts of assists and successful three-point shots, whereas centers excel in rebounds (Sampaio et al., 2006). There are also clusters of a few players, such as “one-of-a-kind,” which are individuals with outstanding talent and statistics, For example, James Harden and Russell Westbrook, who perform well in a variety of statistical data (Alagappan, 2012; Lutz, 2012). While these players are not necessarily guaranteed to win (Lutz, 2012), they are key players that every team pursues. Stated differently, these player clusters might lack representativeness as not all teams can boast of having such players. Consequently, the delineation of players according to roles, rather than rigid positions, facilitates adaptability and flexibility in response to team strategies and individual proficiencies (Lorenzo et al., 2019; Zhang et al., 2019). In the course of a game, adept teams swiftly modify their successive offensive maneuvers in response to varying defensive shifts, whereas alterations in defensive strategies minimally affect offensive actions (Christmann et al., 2018). Hence, characterizing player roles through play-type data will better mirror a player’s skill set and designated role.

An analysis of the offensive role composition in different teams demonstrated significant variations among three distinct offensive role categories, all falling within the classification of big players (N10, N13, F4). Prior studies on player classification consistently demonstrate the relatively low number of big men. Furthermore, the rarity of big men with specific skills like long-range shooting or proficiency in the low post hampers their availability on most teams (Çene, 2018). Consequently, teams without such players must adjust their tactics or select alternative types of big men as substitutes (Wang et al., 2022). The logistic regression results revealed a positive impact of the two offensive roles (N6 and N13) on a team’s advancement to the semi-finals. Consistent with previous studies on play types, successful teams were found to exhibit more cuts, low-post offense, spot-up shots, and free throws (Zukolo et al., 2019; Matulaitis and Bietkis, 2021), which aligns with the characteristics of the two identified offensive roles. Furthermore, the significance of the off-ball movement was emphasized. Additionally, offensive rebounding emerged as a crucial aspect of performance. Offensive rebounding creates secondary scoring opportunities and diminishes the opponent’s chances of transitioning into offense (Zukolo et al., 2019). Ball handlers play a pivotal role within the team, as over half of the set-offense are initiated through ball screens (Gómez et al., 2015; Vaquera et al., 2016). However, research has indicated that lineups with more than two high-usage ball handlers tend to underperform, decreasing team efficiency (Kalman and Bosch, 2020; Wang et al., 2022). The same finding was also seen in the study by Lutz et al. who noted that ball handlers have a negative impact on a team’s point differential (Lutz, 2012). Transition offense, while efficient, does not exhibit significant correlations with game outcomes, likely due to its relatively small proportion within overall gameplay (Selmanovi, 2015; Suárez-Cadenas et al., 2016; Matulaitis and Bietkis, 2021). Consequently, players specializing solely in transition offense and spot-up shooting are considered highly interchangeable. Moreover, players proficient in scoring from screens (N11) heavily rely on the team’s ability to create space for them and face heightened defensive pressure (McIntyre et al., 2016). Notably, off-ball screens and hand-offs contribute the least to scoring; losing teams tend to have more attacks (Suárez-Cadenas et al., 2016; Zukolo et al., 2019).

Finally, our study indicates that the offensive role of foreign players has no substantial impact on team performance. These findings align with prior research that found no significant difference in efficiency between foreign players of top teams and average (non-top) teams (Ozmen, 2012; Wang et al., 2022). Conversely, native players of top teams are more efficient than native players of teams outside the top tier. Native players in the CBA league exhibit distinct offensive characteristics, with spot-up, transition, and cutting actions comprising a significant portion of their gameplay. These offensive strategies prioritize teamwork and have been proven effective in several studies (Christmann et al., 2018; Zukolo et al., 2019; Matulaitis and Bietkis, 2021). However, they rely on teammate support for execution, making these offensive roles often perceived as scoring beneficiaries (Whitehead, 2017b; Zukolo et al., 2019). In contrast, pick-and-roll ball handlers, isolation, and post-ups were more prevalent among foreign players. While some studies disagree on the efficiency of these playing styles (Selmanovi, 2015; Christmann et al., 2018; Zukolo et al., 2019), they tend to emphasize individual ability. Consequently, these offensive roles are regarded as scoring creators (Whitehead, 2017b; Zukolo et al., 2019), particularly in teams lacking strong scoring capabilities, where the role of these players becomes even more vital (Arcidiacono et al., 2017). Conversely, foreign players have demonstrated superior talent to domestic players in the league (Ozmen, 2012; Özmen, 2019; Gasperi et al., 2020). Due to the distinct playing time regulations concerning foreign players in the CBA league, teams are constrained to field a single foreign player during each quarter. This stipulation has led most teams to prioritize the scoring ability of these foreign recruits. Lutz et al. pointed out in their study that some player classifications such as aggressive bigs, big bodies, and perimeter scorers, have a negative impact on the game score difference (Lutz, 2012). Also, it was mentioned in their study that big players are generally unfavorable to the outcome of the game (Lutz, 2012), which diverges from our findings. Our explanation is that similar to the roles of native and foreign players in other leagues, native players play a secondary role in their team’s offense, relying on their teammates to create chances while focusing more on defense and other secondary responsibilities and playing a lesser role in the creative aspects of the game (Guimarãe et al., 2018; Özmen, 2019); while some studies have also pointed out that big players who focus on defense have a positive impact on the outcome of the game (Lutz, 2012; Wang et al., 2022), which support our research findings. These insights prompt coaches to recognize that with all teams having the funds to recruit talented foreign players, it is vital to develop native players, especially big men with a great sense of teamwork and defensive ability.

## 5. Limitations and future directions

This study has certain limitations. Firstly, similar to other studies employing clustering for player role classification, the final clustering results are inherently subjective, leading to the possibility of misclassifying players into different roles, which is an unavoidable issue in clustering studies. Secondly, basketball is a multifaceted game with various dimensions, where player roles can significantly fluctuate depending on team strategies, game situations, and opponent circumstances. This study solely analyzed players’ offensive types while neglecting relevant defensive metrics. Some players renowned for their defensive prowess may have been overlooked, potentially oversimplifying players’ roles and disregarding the diversity of their on-court contributions. Lastly, when studying the impact of offensive roles on team performance, we solely employed the number of players as the independent variable due to sample size limitations; other variables were not considered, such as players’ playing time and efficiency values across different play types, which constrained our ability to investigate further the influence of players’ roles on team performance.

Regarding future research directions, several areas warrant exploration. First, player roles evolve in response to coaching decisions, formation adjustments, and player development. Investigating the patterns and trends of these role changes is one of the areas we intend to explore. Second, incorporating a larger sample size and additional influential variables concerning the impact of player roles on team performance would enhance model stability, thereby facilitating the further investigation of the matter. Lastly, future research should integrate advanced machine learning techniques like deep or reinforcement learning to capture player roles’ intricate relationships and temporal dynamics. These approaches are adept at handling large-scale data, identifying complex patterns and dependencies, and yielding a more nuanced comprehension of offensive roles.

## 6. Conclusion

In this study, we utilize play-type data with interaction information to categorize native and foreign players in the CBA league into 14 and five distinct offensive roles, respectively, employing a clustering approach. These results provide a unique perspective for understanding and observing the player’s role. Further research found that big players made a difference in team composition. The offensive roles of two categories of native players, whose attributes emphasized both teamwork and off-ball movement, showed a significant influence on team performance. In contrast, no such impact was found for foreign players. Consequently, basketball coaches and managers should consider optimizing the composition of individual player clusters during the team-building process and prioritize the development of native players.

## Data availability statement

The original contributions presented in the study are included in the article/Supplementary material, further inquiries can be directed to the corresponding author.

## Ethics statement

Ethical review and approval was not required for the study on human participants in accordance with the local legislation and institutional requirements. Written informed consent from the patients/participants or patients/participants' legal guardian/next of kin was not required to participate in this study in accordance with the national legislation and the institutional requirements.

## Author contributions

RC: Data curation, Software, Visualization, Writing – original draft. MZ: Conceptualization, Funding acquisition, Methodology, Supervision, Writing – review & editing. XX: Methodology, Software, Writing – review & editing.

## Funding

The author(s) declare that no financial support was received for the research, authorship, and/or publication of this article.

## Conflict of interest

The authors declare that the research was conducted in the absence of any commercial or financial relationships that could be construed as a potential conflict of interest.

## Publisher’s note

All claims expressed in this article are solely those of the authors and do not necessarily represent those of their affiliated organizations, or those of the publisher, the editors and the reviewers. Any product that may be evaluated in this article, or claim that may be made by its manufacturer, is not guaranteed or endorsed by the publisher.

## Footnotes

^1^ https://synergysports.com/sport/basketball/
